# Absence of *Clostridium difficile* stool carriage in asymptomatic volunteers

**DOI:** 10.1186/1753-6561-5-S6-P183

**Published:** 2011-06-29

**Authors:** M Hell, K Sickau, G Chmelizek, JM Kern, M Maass, S Huhulescu, F Allerberger

**Affiliations:** 1 Dep.of Hospital Epidemiology and Infection Control, University Hospital Salzburg, Salzburg, Austria; 2 Institut of Hygiene, microbiology and infectious diseases, University Hospital Salzburg, Salzburg, Austria; 3 National Reference Laboratory for Clostridium difficile, Austrian Agency for Health and Food Safety (AGES), Vienna, Austria

## Introduction / objectives

*Clostridium difficile*Â is considered a leading cause of hospital-acquired diarrhea. Currently there areÂ published case-reports of symptomatic Health-Care-Workers (HCW)Â and one report demonstratingÂ transmission of C. diff from patient to HCW. Therefore, we initiated a prospective study to evaluate the prevalence of asymptomatic *C. difficile* stool carriage among healthcare workers at a single university hospital comparing themÂ to non-healthcare workers to asses the risk for HCW’s acquiring Clostridium difficile.

## Methods

The study population consisted of 113 healthy HCW’s of clinical departments with a high incidence ofÂ CDI in inpatients. The 128 controls were taken from the administration department of a Food Company and from frozen stool samples of healthy subjects from a colon cancer screening program. Both groups were comparable in age-and sex-distribution. From April to July 2010, in total 241 stool specimens were tested for toxigenic culture of C diff.. 51% of stool samples (58/113) of the study population and all control-samples (n=128) were confirmed by broth enrichment technique at the National Reference Laboratory for *C. difficile* in Vienna.

## Results

Both investigated study-groups (n-total = 241) were negative for Clostridium difficile by both culture techniques (direct plating and broth enrichment method).

## Conclusion

We conclude, therefore, that healthy HCWs are probably not at risk for aquiring C diff spores from contacts with CDI-patients. They are themselves no risk for spreading C. diff spores in health-care facilities. Data about C.diff carriage in the community (up to 3%) demonstrates a possible overestimation.

## Disclosure of interest

None declared.

